# Recurrent Desmoid Tumor of the Neck: A Case Report of a Benign Disease with Aggressive Behavior

**DOI:** 10.1155/2018/6573587

**Published:** 2018-11-28

**Authors:** Dionisios A. Klonaris, Alexander D. Karatzanis, Stylianos G. Velegrakis, Eleni D. Lagoudakis, Emmanuel P. Prokopakis, George A. Velegrakis

**Affiliations:** ^1^Department of Otorhinolaryngology, University of Crete Medical School, Heraklion, Crete, Greece; ^2^Department of Pathology, University of Crete Medical School, Heraklion, Crete, Greece

## Abstract

We present a case of a desmoid tumor recurrence in a patient with a history of a resected desmoid tumor of the right neck area with free surgical margins six months earlier. The neoplasm was found to invade the parapharyngeal space, and wide excision was performed including most of the sternocleidomastoid muscle (SCM), the thrombosed internal jugular vein (IJV), and the infiltrated spinal accessory nerve (SAN). The histopathologic findings displayed free microscopic margins, with close margins at the site of the parapharyngeal space extension. After 3 months, there was no sign of tumor recurrence. After 6 months, local tumor recurrence was identified on clinical examination and imaging. The decision of the Oncology Board was further treatment with radiotherapy (RT). Response to treatment was satisfactory, and the patient was on close follow-up for twelve months. Desmoid tumors are very rare benign neoplasms of mesenchymal origin with negligible mortality but high morbidity, due to their high recurrence rates, local tissue infiltration, and unpredictable disease course and response to treatment. No universally acceptable treatment protocols have been introduced to date. Appropriate patient counseling and close follow-up are warranted in all cases.

## 1. Introduction

Desmoid tumors, also known as “well-differentiated nonmetastasizing fibrosarcoma,” “Grade I fibrosarcoma,” and “aggressive fibromatosis,” are very rare benign neoplasms of mesenchymal origin [1]. They arise from musculoaponeurotic structures throughout the body. Estimated overall incidence is 2–4 cases per million, and appearance in the head and neck region concerns only a small minority of cases. Despite their benign nature and typically slow growth rate, these tumors exhibit a locally aggressive infiltrative behavior. Thus, they frequently invade nearby structures and show a tendency for local recurrence [1]. In this report, we present a case of a desmoid tumor of the neck with locally aggressive behavior requiring combined modality treatment for disease control. Informed consent was received from the patient for the publication of this manuscript. Case presentation is followed by a concise review of the literature.

## 2. Case Report

**Figure 1:**
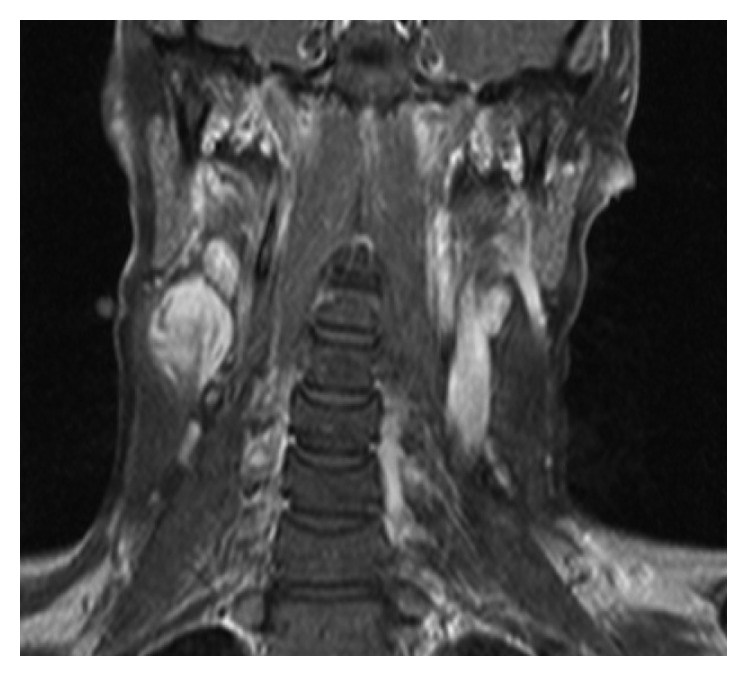
Coronal T1-weighted fast spin-echo (T1FSE) MRI depicting the tumor as an enhancing mass at the cephalic part of the right sternocleidomastoid muscle.

A 22-year-old Caucasian female, nulligravida, presented to our institute (a tertiary referral center) complaining of a slowly growing painful mass at the right lateral neck. Past medical history included a resected desmoid tumor with free surgical margins from the same region six months ago in another center (Figure 1). No other comorbidities were reported. Her family history included mother with systemic lupus erythematosus. Physical examination revealed a hard, tender, palpable mass over the upper half of the right SCM, painful head rotation, and right upper extremity extension but no other sensory deficits or motion restrictions.

**Figure 2:**
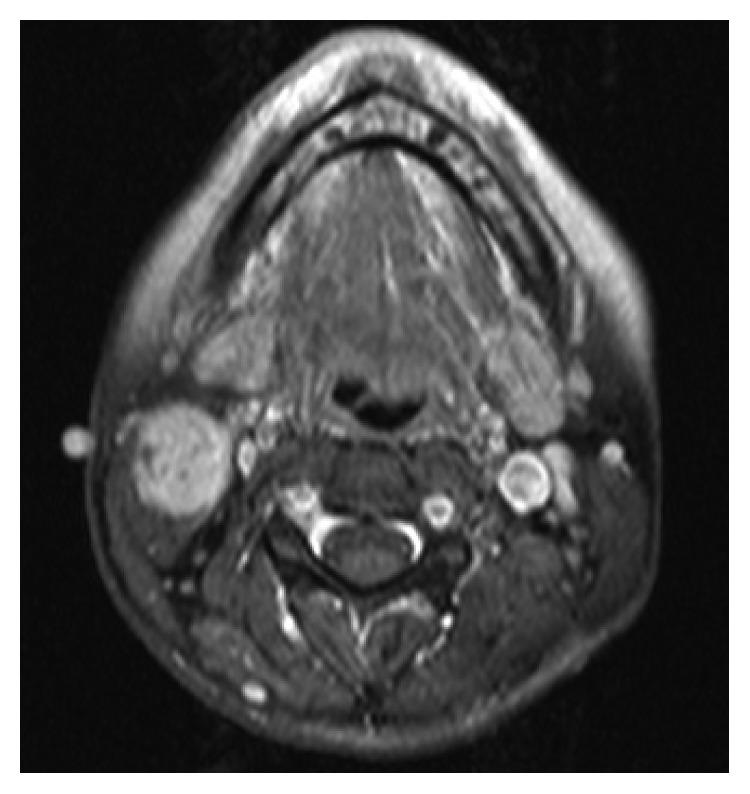
Axial T1-weighted fast spin-echo (T1FSE) MRI showing an enhancing mass indicative of local tumor recurrence following surgery.

MRI revealed an enhancing mass at the cephalic third of the SCM, in close contact with the right IJV, with no signs of vessel infiltration (Figure 2). No pathologic cervical lymph nodes were detected by MRI and ultrasound tomography.

**Figure 3:**
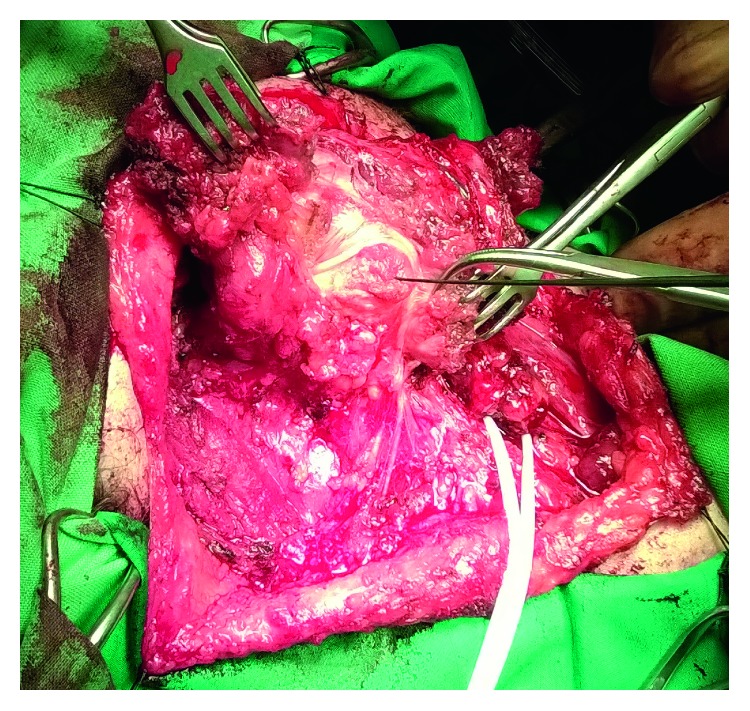
Surgical excision of the desmoid tumor.

**Figure 4:**
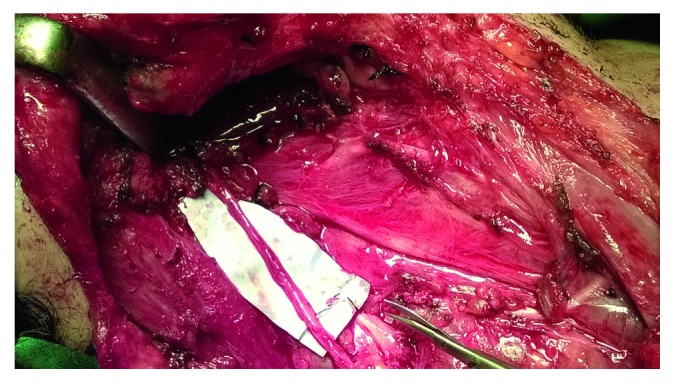
End-to-end anastomosis of the stumps of the spinal accessory nerve after macroscopic complete resection of the desmoid tumor.

Given her past medical history, imaging findings, and clinical presentation, the patient was scheduled two months later for surgical excision of the tumor recurrence under general anesthesia. Access to the surgical field was via an oblique right lateral neck incision. The neoplasm was found to originate from the upper portion of the SCM, extending to the parapharyngeal space, and infiltrating the SAN (Figure 3). A wide excision was performed, including the upper two-thirds of the SCM, the tumor extension to the prestyloid parapharyngeal space, the stylohyoid muscle, and part of the styloid process. The completely thrombosed ipsilateral IJV was ligated and excised. Intraoperatively, it was deemed impossible to dissect the SAN free from the neoplasm and so it had to be sacrificed. However, remaining length of the nerve was satisfactory, and a microsurgical end-to-end anastomosis was performed (Figure 4). A close suction drain was placed, and the wound was closed in layers. Patient recovery from the operating room was without any incidents.

The patient was discharged on the second postoperative day in good condition. The range of right upper extremity extension was limited, and the patient was referred for physiotherapy.

**Figure 5:**
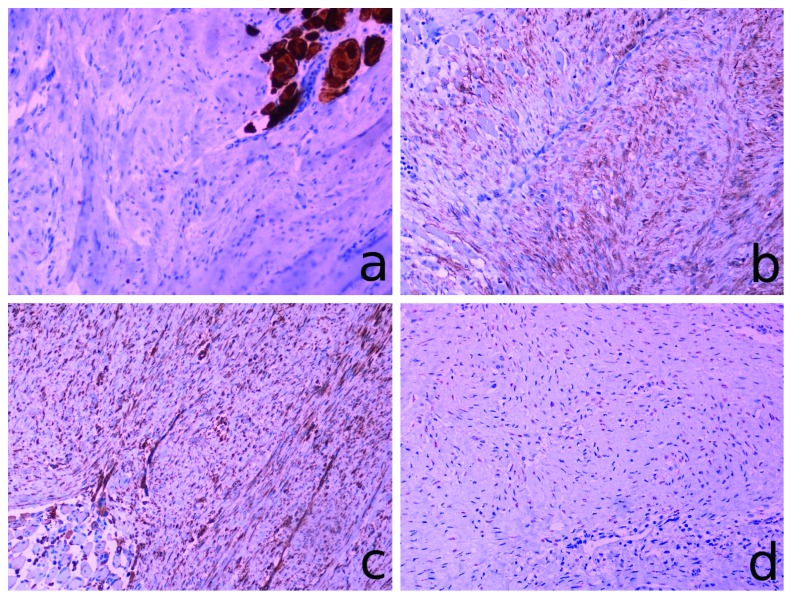
Immunohistochemical staining of the biopsy specimen: (a) desmin; (b) smooth muscle actin; (c) vimentin; (d) *β*-catenin.

Microscopically, the tumor consisted of fascicles of uniform elongated fibroblast-like cells, surrounded and separated by abundant collagen, with no cell-to-cell contact. Neoplastic cells had sharply defined nuclei with one or more delicate nucleoli and poorly defined cell borders often merging with the extracellular collagen. Regenerative multinucleated skeletal muscle cells were found in the periphery of the lesion. Mitotic activity was typically low (2 mitoses per 10 HPFs) without atypical mitoses. Immunohistochemically, neoplastic cells stained vimentin (Figure 5), smooth muscle actin (Figure 5), and desmin (Figure 5) and were negative for CD34. *β*-Catenin nuclear staining was also present (Figure 5). The index of proliferation Ki-67 was 1-2%. Focally close excision margins were noted at the parapharyngeal border of the biopsy specimen.

On the 3-month postoperative follow-up, no clinical signs of tumor recurrence were noted, and the right upper extremity mobility was satisfactory, indicating successful anastomosis of the SAN. Unfortunately, on the 6-month follow-up, local pain and swelling indicated new tumor recurrence which was confirmed by MRI. The case of our patient was brought to the Oncology Board. Unanimously, it was agreed that a wait-and-see policy was not a sound option and some kind of treatment should be administered since the tumor recurred within six months. Based on imaging findings, excellent performance status of the patient, and our experience as a tertiary referral center on head and neck surgery, excision of the new tumor recurrence was considered as the primary treatment modality. RT was also recommended, but given its long-term side-effects and the proximity of neural structures to the tumor bed, it was considered as an alternative option. Cytotoxic agents, tyrosine kinase inhibitors, and hormonal therapy were considered but discarded, given the reproductive age of our patient and the absence of contraindications for surgery or RT. The patient declined the option of further surgery but elected for RT and received 54 Gy of external beam RT over six weeks. Treatment was well tolerated. After the completion of RT, complete clinical remission of the disease was noted. No evidence of recurrence has been detected on clinical evaluation and imaging studies after twelve months of follow-up.

## 3. Discussion

According to the location of occurrence, desmoid tumors are classified into intra-abdominal, abdominal, and extra-abdominal types [2]. About 30% are extra-abdominal and only 10–25% of these develop in the head and neck area [2]. Tumor etiology is obscure although multiple mechanisms have been implicated. A history of prior trauma has been associated with their occurrence although their monoclonal nature does not support this hypothesis [3]. Observations on pattern of behavior during pregnancy, menopause, and contraceptive and tamoxifen administration suggest a sex hormone dependency. Cyclooxygenase-2 has been shown to modulate fibroblast proliferation on desmoid tumors [3]. Some cases of desmofibromatosis are associated with familial adenomatous polyposis in the hereditary Gardner syndrome, with a relative risk of about 850 times higher than that of the general population [3].

Desmoid tumors present as circumscribed, firm overgrowths of fibrous tissue arising from virtually any area with a connective tissue component, showing propensity to infiltrate surrounding tissues and occasionally encase blood vessels and nerves, without apparent deep invasion [4]. Histologically, the neoplasm is composed of mature spindle-form fibroblasts with a rich collagenous component, with a variable mitotic index but no evidence of cellular anaplasia or abnormal mitoses [4]. Diagnosis is almost always established by open or core needle biopsy since fine-needle aspiration cytology does not reliably yield diagnostic results [5, 6]. Computerized tomography and/or magnetic resonance imaging (MRI) are usually employed for tumor extension assessment and preoperative planning [5]. Patients with head and neck desmofibromatosis usually present with a nontender mass, in addition to sensory or motor deficits when nerve or muscular structures are involved [7]. Other symptoms, like dysphagia or hoarseness, may appear when the mass compresses the upper aerodigestive tract [7]. These neoplasms have zero metastatic potential, even though malignant transformation has been very rarely described [8]. Tumor growth speed is typically slow; however, rapid progression of the disease may be seen occasionally [6, 8]. Differential diagnosis should always include metastasis of unknown origin, as well as more unusual diseases such as neurofibroma, fibromatosis colli, congenital generalized or solitary fibromatosis, and diffuse infantile fibromatosis [8, 9].

No universally acceptable therapeutic protocols for extra-abdominal desmoid tumors have been introduced to date. Most information regarding this rare entity originates from retrospective studies and sporadic case reports. It should be noted that while the vast majority of patients are adults, it does not spare the pediatric population [10]. As such, vigilance is required and the disease should always be included in the differential diagnosis of a child with a lateral neck tumor.

Surgical excision of the disease has long been the mainstay of treatment. Tumors of the head and neck pose considerable challenges for the surgeon due to close proximity to vital structures. In addition, treatment-induced morbidity and unsatisfactory local control rates, along with spontaneous regression observed occasionally, have put aggressive surgical treatment into question. Finally, there are no established prognostic factors that can be employed for decision-making on treatment modality [11].

Significance of surgical margin status for this neoplasm is a matter of debate. When technically feasible, complete tumor resection with negative microscopic margins is the optimal treatment choice. The importance of microscopic positive margin status with regard to local recurrence rates is still unclear; the presence of microscopic disease has not been shown to affect long-term disease-free survival [12, 13]. Moreover, peripheral nerve involvement is not uncommon. Although it is usually feasible to preserve these structures during surgery [12], this was not possible in our case.

Further research is required to decrypt the molecular mechanisms underlying desmoid tumor genesis and progression. Acquiring such knowledge may lead to better patient selection, superior treatment, and optimal assessment of prognosis of each case. According to the current literature, CTNNB1 (41A, 45F, 45P), *β*-catenin, and APC locus gene mutations have been implicated with tumor pathogenesis and disease prognosis [14], but there is still no final verdict on the molecular determinants of desmofibromatosis.

Multiple conservative treatment modalities have been recommended and used in different settings. Radiotherapy has been applied primarily for inoperable or inaccessible disease. In addition, it has been used as adjuvant treatment in cases with positive surgical margins and a higher risk of local recurrence. Noncytotoxic regimens like nonsteroidal anti-inflammatory drugs (NSAIDs) and/or tamoxifen have been employed as first-line medical treatment. Cytotoxic agents including anthracycline-containing regimens, doxorubicin, vinblastine, methotrexate, and hydroxyurea have also been employed in cases where surgery and/or radiotherapy are contraindicated, producing variable but, in general, satisfactory results. Response rates to tyrosine kinase inhibitors (imatinib) administration have been shown to be subpar, and this choice should be reserved for cases that have failed other treatment options [3, 10, 14, 15]. Finally, in selected cases, a period of watchful waiting until clinically significant recurrence develops may be the indicated management strategy [3].

## 4. Conclusion

Desmofibromatosis of the head and neck has negligible mortality but high morbidity, due to high recurrence rates and unpredictable biological behavior and response to treatment. A high level of suspicion, appropriate patient counseling, and close follow-up are warranted in all cases [16].
